# Characterization of the Hot Anode Paste Compaction Process: A Computational and Experimental Study

**DOI:** 10.3390/ma12050800

**Published:** 2019-03-08

**Authors:** Hicham Chaouki, Stéphane Thibodeau, Mario Fafard, Donald Ziegler, Houshang Alamdari

**Affiliations:** 1 Department of Civil and Water Engineering, NSERC/Alcoa Industrial Research Chair MACE3 and Aluminium Research Centre—REGAL, 1065 avenue de la Medecine, Laval University, Quebec, QC G1V 0A6, Canada; mario.fafard@gci.ulaval.ca; 2 ABB Inc., Measurement & Analytics Business Unit, 3400 Rue Pierre-Ardouin, Québec, QC G1P 0B2, Canada; stephane.thibodeau@ca.abb.com; 3 Alcoa Primary Metals, Alcoa Technical Center, 859 White Cloud Road, New Kensington, PA 15068, USA; donald.ziegler@alcoa.com; 4 Department of Mining, Metallurgical and Materials Engineering, NSERC/Alcoa Industrial Research Chair MACE3 and Aluminium Research Centre—REGAL, 1065 avenue de la Medecine, Laval University, Quebec, QC G1V 0A6, Canada; houshang.alamdari@gmn.ulaval.ca

**Keywords:** green anode paste, compaction test, nonlinear viscoplastic constitutive law, finite element method, X-ray tomography

## Abstract

The aim of this work is to model and characterize green anode paste compaction behavior. For this purpose, a nonlinear viscoplastic constitutive law for compressible materials, based on the finite strain theory and the thermodynamic framework, was used. An experimental study was carried out to characterize axial and radial behaviors of the anode paste. To this end, simple compaction tests using a thin steel instrumented mold were performed at a temperature of 150 °C. Results of these experiments brought out the nonlinear mechanical behavior of the anode paste. Furthermore, they showed the importance of its radial behavior. The constitutive law was implemented in Abaqus software through the user’s material subroutine VUMAT for explicit dynamic analysis. An inverse analysis procedure for material parameters identification showed that the model predicts compaction tests results with a good agreement. In order to assess the constitutive law predictive potential in situations involving density gradients, compaction tests using complex geometries such as slots and stub holes were carried out. Finite element simulation results showed the ability of the model to successfully predict density profiles measured by the X-ray tomography.

## 1. Introduction

The Hall–Héroult process, used for aluminum production, is characterized by several complex multiphysical phenomena such as thermo-electromechanical, electrochemical, and magneto-hydrodynamic problems. Although this process has undergone major technical developments in recent decades, it remains a high-energy-consumption process and represents a non-negligible source of greenhouse gas emissions [1]. In this context, considerable efforts are invested in optimizing this process. One of the promising solutions could be control and the improvement of the green anode quality, which substantially affects the electrolysis process efficiency.

The green anode is formed either by a compaction or a vibrocompaction process. The carbon paste used is composed of carbon aggregates, fine particles, air voids, and the coal tar pitch that acts as a binder matrix. The presence of complex geometries in the anode such as stub holes and slots generally leads to significant density gradients that could alter the anode quality [2]. Hence, the optimization of the anode-forming process is of great interest for the aluminum industry. Nevertheless, taking into account the high number of experimental tests required to improve the anode quality and the resulting costs, it seems that modelling and the numerical simulation could be appropriate and powerful tools that might successfully help to achieve this challenge.

To the best knowledge of the authors, research works on the anode forming process are few and the problem is still obviously ill-understood. In [2], a simple dynamic model was developed to simulate the anode behavior during vibrocompaction. In [3], the vibrocompaction of the anode was experimentally studied at laboratory scale and a dynamic model taking into account the anode’s stiffness evolution was proposed. However, in these two works the rheological behavior of the anode was not investigated. In [4,5], the discrete element method (DEM) was used to study the anode compaction. Even though this approach is suitable to investigate the mechanical behavior of the anode paste constituents, it cannot be applied straightforwardly at the macro-mechanical scale. Furthermore, the combination of the pitch as a binder matrix with aggregates is still a challenging problem when dealing with this method. In [6], a viscoplastic material model, inspired from a work on the compaction of asphalt mixtures [7], was used to characterize the compaction of the anode paste. In this work, the authors investigated only the anode axial behavior since a rigid mold was used in their experimental study.

From another standpoint, the anode paste’s composition is similar to materials like asphalt mixtures and ramming pastes. In this context, several constitutive laws involving elastoplastic and viscoplastic behaviors for a single phase were developed [8,9,10,11]. Due to the compressibility nature of such materials, material parameters related to these constitutive laws evolve, among others, as functions of the strain rate [9], aggregates microstructural properties [10], and the density evolution [7].

This work aims to investigate anode paste’s axial and radial behaviors during the compaction process. To this end, an experimental study using a steel thin-walled mold was carried out at 150 °C. A viscoplastic material model based on the finite strain theory proposed in an earlier study [6] was considered. An inverse identification procedure shows that the considered material model reproduces experimental trends for both axial and radial directions. Furthermore, finite element simulation results on the density profile of compaction tests involving complex geometries, such as slots and stub hole, are compared to experimental results provided by X-ray computed tomography.

The paper is organized as follows. In Section 2, the viscoplastic constitutive law used in the present study is briefly described. The experimental study and the subsequent results are presented in Section 3. Section 4 is dedicated to the finite element simulation results. In the first step, the inverse identification procedure that was followed to find an optimal set of the constitutive law material parameters, is presented. In the second step, finite element simulation predictions for compaction tests with complex geometries, are compared to experimental trends.

## 2. Constitutive Law

In this section, the main steps for the development of the constitutive law are described. The reader is referred to Reference [6] for a detailed mathematical description of the model. The carbon paste is considered as a single phase isotropic compressible material undergoing finite strains. Using the thermodynamic framework and the concept of the intermediate stress-free configuration [12], the model is built up in two steps: (i) an expression of the stress state is obtained by considering the Clausius–Duhem inequality and (ii) a dissipation potential is proposed to characterize the evolution of the intermediate stress-free configuration.

**Figure 1:**
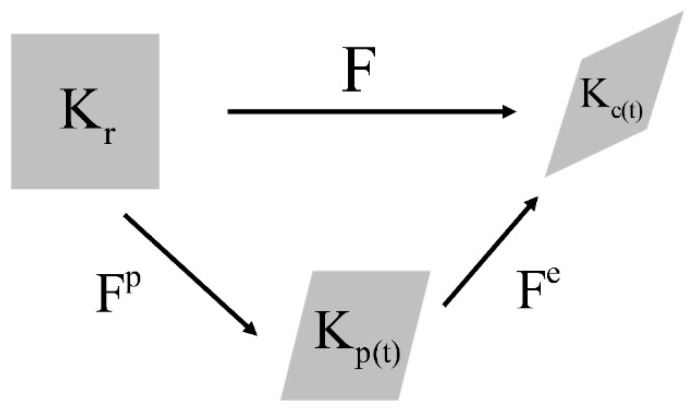
Multiplicative decomposition of the deformation gradient tensor.

Let $k_{r}$ denote the material reference configuration. Assuming that the material is subjected to a motion of deformations χ, the reference configuration is mapped at each time t to the current configuration $k_{c(t)}$ (Figure 1). Consider the following kinematic tensors: the deformation gradient tensor $F=\nabla_{X}\chi$, the right (the left) Cauchy–Green stretch tensor $C=F^{T}.F$ ($B=F.F^{T}$), the velocity gradient tensor $L=\nabla_{x}v=\dot{F}.F^{-1}$ and the rate of deformation tensor D=sym[L]. To take into account the irreversible behavior of the anode paste, the intermediate stress-free configuration $k_{p(t)}$ is introduced. Consequently, the deformation gradient can be decomposed as (Figure 1):

(1) $$F=F^{e}.F^{p},$$

$F^{e}$ is the deformation gradient’s component accounting for the elastic response of the material that holds between configurations $k_{p(t)}$ and $k_{c(t)}$; the tensor $F^{p}$, mapping the configuration $k_{r}$ to the intermediate configuration $k_{p(t)}$, accounts for the permanent part of the deformation process.

Kinematic tensors associated with the intermediate configuration $k_{p(t)}$ are introduced: the right (the left) Cauchy–Green stretch tensor $C^{e}={\left(F^{e}\right)}^{T}.F^{e}\left(B^{e}=F^{e}.{\left(F^{e}\right)}^{T}\right)$, the velocity gradient tensor $L^{p}=\dot{\overset{⌢}{F^{p}}}.{\left(F^{p}\right)}^{-1}$, and the rate of deformation tensor $D^{p}=sym\left[L^{p}\right]$.

Assuming that the anode compaction process is carried out under an isothermal process, the Clausius–Duhem inequality is expressed as [13]:

(2) $$0\leq \Phi =\sigma :L-\rho \dot{\Psi },$$

Φ represents the mechanical energy dissipation, σ is the Cauchy stress tensor, ρ is the density, and Ψ is the specific Helmholtz free energy.

In this work, the Helmholtz free energy is assumed as a function of scalar invariants:

(3) $$\Psi =\Psi \left(I_{B^{e}},\;{\mathrm{III}}_{B^{e}},\;{\mathrm{III}}_{F^{p}}\right),$$

where

(4) $$\{\begin{array}{ccc} I_{B^{e}} & = & \mathrm{Tr}\left(B^{e}\right)\\ {\mathrm{III}}_{B^{e}} & = & \det\left(B^{e}\right)\\ {\mathrm{III}}_{F^{p}} & = & \det\left(F^{p}\right) \end{array}\;.$$

The third scalar invariant, ${\mathrm{III}}_{F^{p}}$, of the tensor $F^{p}$ is considered to account for the evolution of the constitutive law’s parameters.

The expression of the mechanical dissipation can be rewritten according to Definition (3) and through the development of the material derivative of the free energy, as follows [6]:

(5) $$\begin{array}{ll} 0\leq \Phi = & \left[\sigma -2\rho \frac{\partial \Psi }{\partial I_{B^{e}}}B^{e}-2\rho {\mathrm{III}}_{B^{e}}\frac{\partial \Psi }{\partial {\mathrm{III}}_{B^{e}}}I\right]:D+\\ & \left[2\rho \frac{\partial \Psi }{\partial I_{B^{e}}}C^{e}+2\rho {\mathrm{III}}_{B^{e}}\frac{\partial \Psi }{\partial {\mathrm{III}}_{B^{e}}}I-\rho {\mathrm{III}}_{F^{p}}\frac{\partial \Psi }{\partial {\mathrm{III}}_{F^{p}}}I\right]:D^{p} \end{array}$$

From Equation (5), one can assume that the material’s stress state is given by:

(6) $$\sigma =2\rho \frac{\partial \Psi }{\partial I_{B^{e}}}B^{e}+2\rho {\mathrm{III}}_{B^{e}}\frac{\partial \Psi }{\partial {\mathrm{III}}_{B^{e}}}I.$$

Therefore, the intrinsic energy dissipation becomes:

(7) $$0\leq \Phi =\rho \left[2\frac{\partial \Psi }{\partial I_{B^{e}}}C^{e}+2{\mathrm{III}}_{B^{e}}\frac{\partial \Psi }{\partial {\mathrm{III}}_{B^{e}}}I-{\mathrm{III}}_{F^{p}}\frac{\partial \Psi }{\partial {\mathrm{III}}_{F^{p}}}I\right]:D^{p}\;.$$

The Cauchy stress tensor (Equation (6)) is expressed relative to the intermediate stress-free configuration which is unknown. To complete the elaboration of the model, a dissipation potential has to be defined with the purpose of characterizing the intermediate configuration evolution. To this end, the potential of the dissipation is assumed to be quadratic and it is defined as [6]:

(8) $$\Phi =\eta \left({\mathrm{III}}_{F^{p}}\right)\left(C^{e}.D^{p}\right):D^{p}$$

The material parameter $\eta \left({\mathrm{III}}_{F^{p}}\right)$ represents the viscosity of the whole anode paste, which evolves during the compaction process.

By comparing Equations (7) and (8) and considering the stress Equation (6), one can show that the intermediate configuration $k_{p(t)}$ is governed by the following differential equation (see [6] for a detailed proof):

(9) $$-\frac{\eta }{2}\left(\overset{•}{\overset{⌢}{B^{e}}}-L.B^{e}-B^{e}.L^{T}\right)=\sigma -\rho {\mathrm{III}}_{F^{p}}\frac{\partial \Psi }{\partial {\mathrm{III}}_{F^{p}}}I.$$

Considering the upper convected Oldroyd derivative of $B^{e}$, defined as:

(10) $$\overset{\nabla }{B^{e}}=\overset{•}{\overset{⌢}{B^{e}}}-LB^{e}-B^{e}L^{T},$$

one can conclude that the intermediate stress-free configuration is characterized through the following equation:

(11) $$\overset{\nabla }{B^{e}}=-\frac{2}{\eta \left({\mathrm{III}}_{F^{p}}\right)}\left(\sigma -\rho {\mathrm{III}}_{F^{p}}\frac{\partial \Psi }{\partial {\mathrm{III}}_{F^{p}}}I\right)\;.$$

For the specific Helmholtz free energy, the neo-Hookean form for a compressible material was adopted [14]:

(12) $$\Psi \left(I_{B^{e}},{\mathrm{III}}_{B^{e}},{\mathrm{III}}_{F^{p}}\right)=\frac{\mu \left({\mathrm{III}}_{F^{p}}\right)}{2\rho_{k_{p(t)}}}\left[I_{B^{e}}-3-\ln\left({\mathrm{III}}_{B^{e}}\right)\right]+\frac{\lambda \left({\mathrm{III}}_{F^{p}}\right)}{8\rho_{k_{p(t)}}}\ln{\left({\mathrm{III}}_{B^{e}}\right)}^{2}.$$

Material functions μ(.) and λ(.) evolve as the anode paste is deformed. They are related to the evolution of the shear modulus and the Poisson ratio, respectively. $\rho_{k_{p(t)}}$ is the density related to the intermediate stress-free configuration $k_{p(t)}$.

Using the stress expression (Equation (6)) and the free energy definition (Equation (12)), the Cauchy stress tensor becomes:

(13) $$\sigma =\frac{\mu \left({\mathrm{III}}_{F^{p}}\right)}{\sqrt{{\mathrm{III}}_{B^{e}}}}B^{e}+\frac{1}{\sqrt{{\mathrm{III}}_{B^{e}}}}\left(\frac{\lambda \left({\mathrm{III}}_{F^{p}}\right)}{2}\ln\left({\mathrm{III}}_{B^{e}}\right)-\mu \left({\mathrm{III}}_{F^{p}}\right)\right)I\;.$$

Expressions used in [7] to define shear and viscosity functions will be considered in this work:

(14) $$\mu \left({\mathrm{III}}_{F^{p}}\right)=\hat{\mu }{\left[1+\lambda_{1}{\left({\mathrm{III}}_{F^{p}}\right)}^{2n_{1}}\right]}^{q_{1}}\;,$$

(15) $$\eta \left({\mathrm{III}}_{F^{p}}\right)=\hat{\eta }{\left[1+\lambda_{2}{\left({\mathrm{III}}_{F^{p}}\right)}^{2n_{2}}\right]}^{q_{2}}\;,$$

where $\hat{\mu },\;\hat{\eta },\;{\left(\lambda_{i}\right)}_{i=1,2},\;{\left(n_{i}\right)}_{i=1,2}$ and ${\left(q_{i}\right)}_{i=1,2}$ are material parameters.

The material function λ(.) appearing in Equation (12) is defined as follows:

(16) $$\lambda \left({\mathrm{III}}_{F^{p}}\right)=\alpha \exp\left(\frac{1-{\mathrm{III}}_{F^{p}}}{\beta }\right),$$

where α and β are material parameters.

In Equations (14)–(16), material parameters are density dependent through the third invariant ${\mathrm{III}}_{F^{p}}$.

## 3. Experimental Study

In order to characterize the mechanical behavior of the anode paste, an experimental study based on compaction tests using a flexible mold wall was carried out at 150 °C. The anode paste consists of coal tar pitch and coke aggregates ranging from fine particles to large aggregates. Fine particles are obtained through ball milling of the calcined coke and the corresponding Blaine number was 4200. The mesh size of aggregates and the pitch content are summarized in Table 1. The paste ingredients were mixed following a procedure developed in [15].

**Figure 2:**
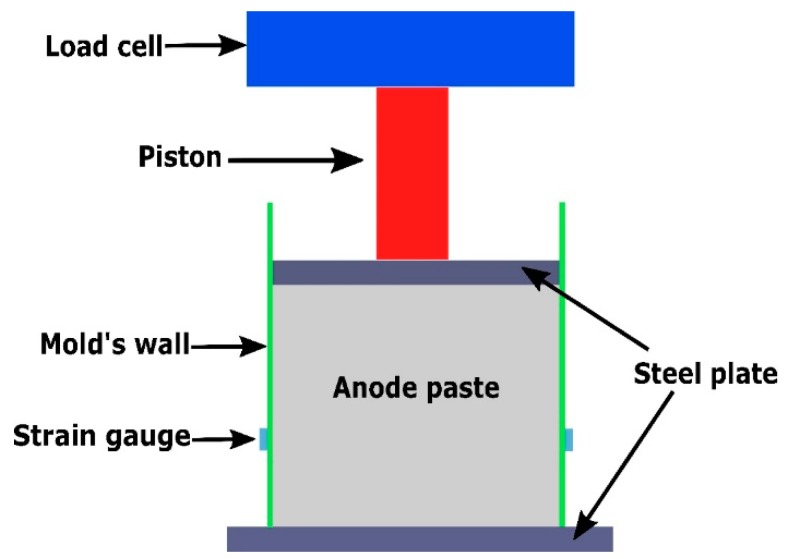
A schematic view of the experimental set-up.

**Figure 3:**
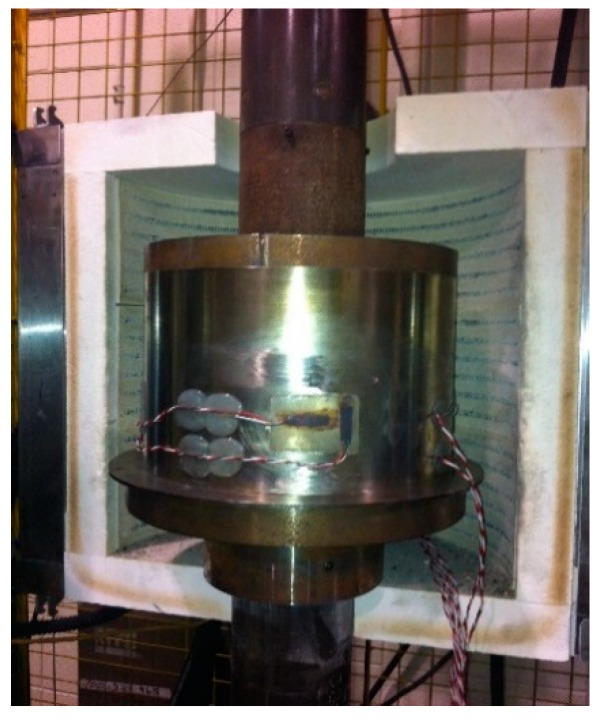
The experimental set-up used for compaction tests.

An experimental setup was developed to carry on compaction tests at high temperature [16]. To this end, a hydraulic press with a cell load of 250 kN, a furnace with controlled temperature and a cylindrical stainless steel mold with thin wall were used (Figure 2 and Figure 3). The mold has a diameter of 254 mm, a height of 140 mm, a wall thickness of 0.356 mm, and is free of joints. Steel plates having a thickness of 30 mm have been fixed on lower and upper pistons of the press to apply a uniform load on the paste surface. During the compression test, the paste undergoes radial and circumferential displacements acting on the mold wall. To measure the paste properties in these directions, the mold wall was instrumented with four strain gauges in the axial direction and four strain gauges in the circumferential direction. Strain gauges were fixed at one-third of the mold height. Tests were performed at 150 °C and under a cross-head speed of 1 mm/s.

**Figure 4:**
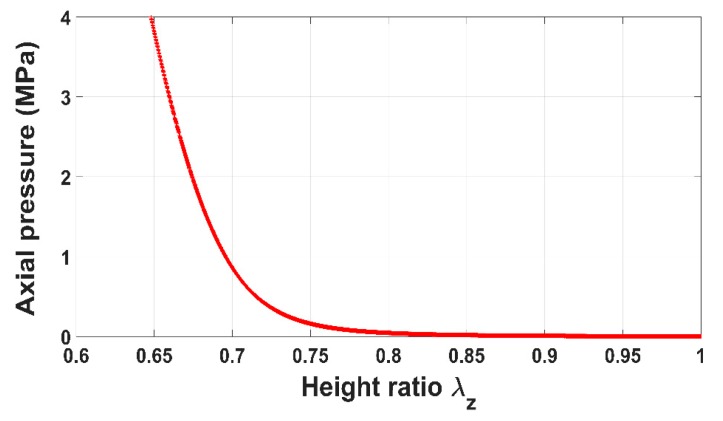
Axial pressure vs. height ratio.

**Figure 5:**
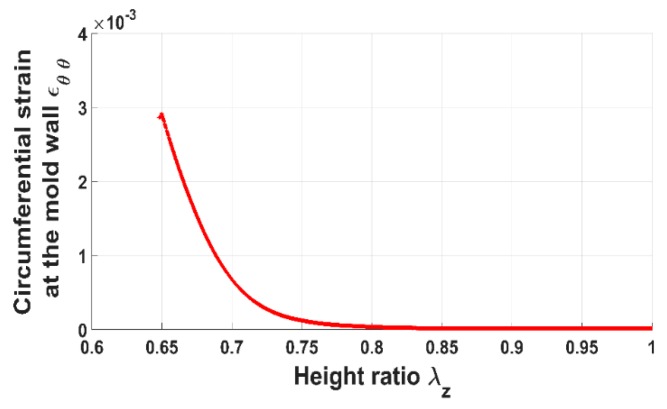
Circumferential strain at the mold wall vs. height ratio.

Figure 4 shows the evolution of the axial pressure acting on the anode paste as a function of the height ratio. The anode paste was compacted up to 35% of its initial volume and the pressure approximately reached 4 MPa. The height ratio represents the stretch of the paste in the direction of the vertical load and it corresponds to the component $F_{zz}$ of the deformation gradient tensor F. Figure 5 illustrates the measured circumferential strain at the mold wall. The strain is the average of strains measured using the four strain gauges. When the anode paste reaches a given level of compaction as about 25% in these experiments, the axial stress and the circumferential strain at the mold wall undergo significant increases. This behavior can be explained by the rigid skeleton formation process. Before this compaction level at which stress and circumferential strain start to evolve significantly, the measured axial stress is negligible due to the high dissipative nature of the compressible paste. Once the skeleton forms, the axial force needed to maintain the compaction process increases and the rigid skeleton acts on the thin mold’s wall, generating the observed circumferential strain behavior.

To characterize the anode paste behavior in the radial direction, the elastic theory of thin shell is used. Considering a thin cylindrical shell subjected to an internal pressure $P_{i}$, strains and pressure are related through the following formulas [17]:

(17) $$u\left(r\right)=\frac{a^{2}P_{i}}{E\left(b^{2}-a^{2}\right)}\left[\left(1-\nu \right)r+\left(1+\nu \right)\frac{b^{2}}{r}\right]$$

(18) $$\varepsilon_{rr}\left(r\right)=\frac{\partial u}{\partial r}=\frac{a^{2}P_{i}}{E\left(b^{2}-a^{2}\right)}\left[\left(1-\nu \right)-\left(1+\nu \right)\frac{b^{2}}{r^{2}}\right]$$

(19) $$\varepsilon_{\theta \theta }\left(r\right)=\frac{u\left(r\right)}{r}=\frac{a^{2}P_{i}}{E\left(b^{2}-a^{2}\right)}\left[\left(1-\nu \right)+\left(1+\nu \right)\frac{b^{2}}{r^{2}}\right]$$

where a and b represent inner and outer radii of the thin shell; E and ν are the Young’s modulus and the Poisson coefficient of the shell; u(r), $\varepsilon_{rr}\left(r\right)$, and $\varepsilon_{\theta \theta }\left(r\right)$ are the radial displacement, radial and circumferential strains for a given radius r∈[a,b], respectively.

Using Equation (19), the internal pressure acting on the mold’s wall is estimated as follows:

(20) $$P_{i}=\frac{E\left(b^{2}-a^{2}\right)}{2a^{2}}\varepsilon_{\theta \theta }\left(r=b\right)$$

where $\varepsilon_{\theta \theta }\left(r=b\right)$ is the measured circumferential strain (Figure 5). The internal pressure corresponds to the stress exerted by the paste on the mold.

The radial displacement of the anode paste that is supposed to be equal to the radial displacement of the mold wall (u(r=a)), is estimated through the Equation (17).

The mold’s elastic properties that were measured using some simple traction tests were equal to E=220 GPa and ν=0.31.

**Figure 6:**
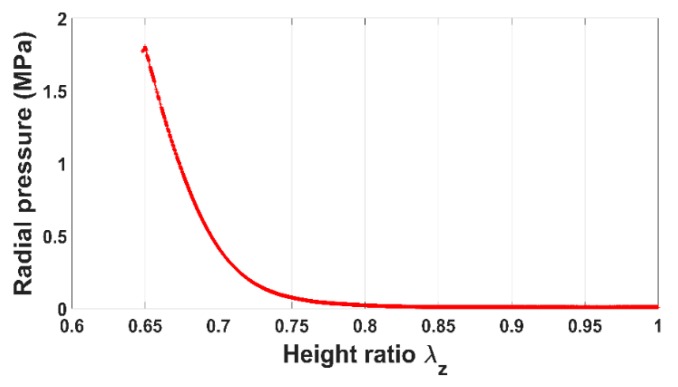
Radial pressure vs. height ratio.

**Figure 7:**
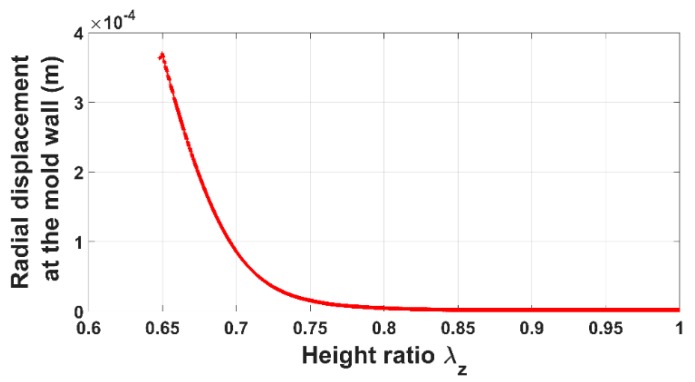
Radial displacement at the mold wall vs. height ratio.

Figure 6 and Figure 7 show the evolution of the radial pressure and the radial displacement of the anode paste at the mold wall. Before reaching the aforementioned compaction level, radial displacement and pressure are negligible. Once the skeleton is established, they evolve significantly. The internal pressure is approximately half of the axial pressure. As a consequence, the anode radial behavior cannot be neglected during the compaction process.

**Figure 8:**
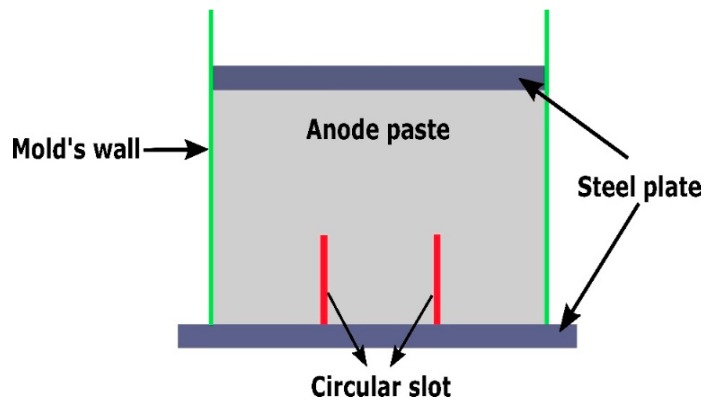
Cross-sectional view of the compaction test with the slot.

**Figure 9:**
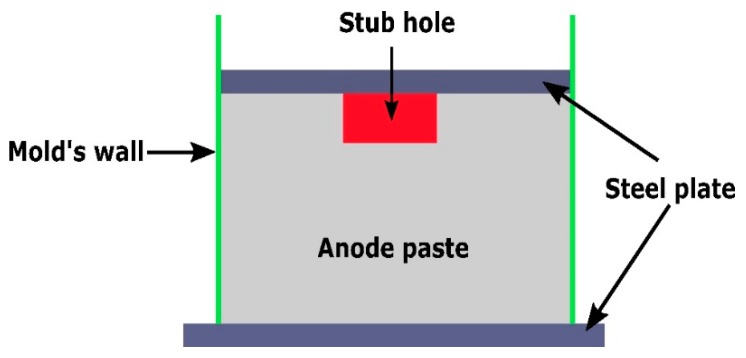
Cross-sectional view of the compaction test with the stub hole.

**Figure 10:**
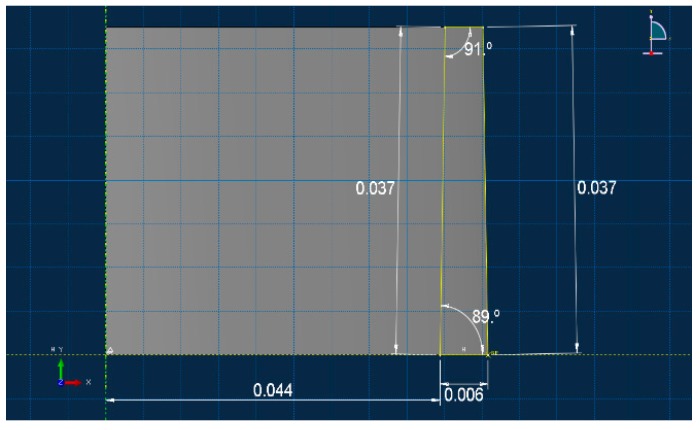
The slot geometry dimensions (m).

**Figure 11:**
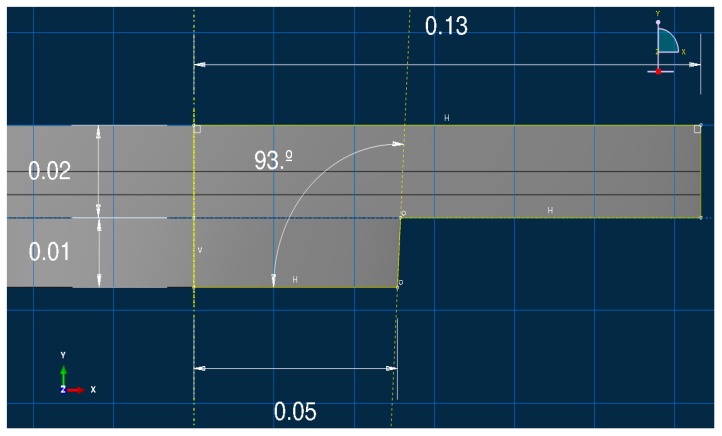
The stub-hole geometry dimensions (m).

**Figure 12:**
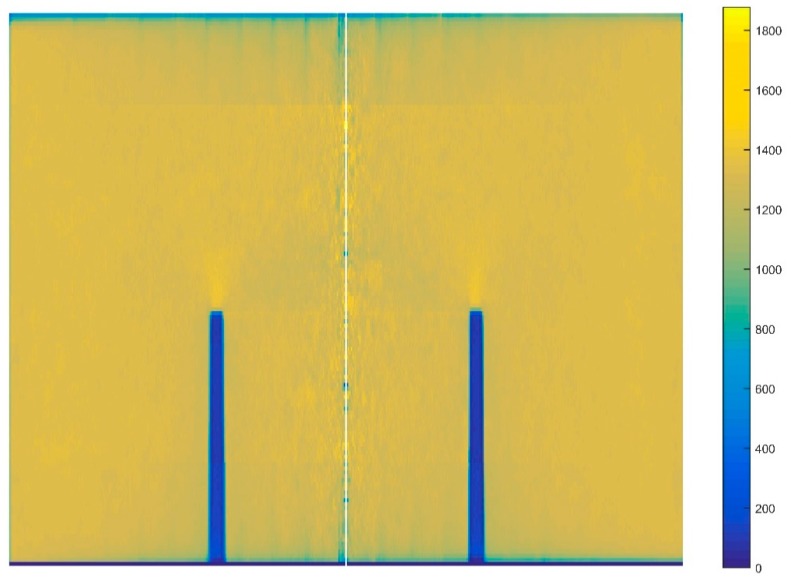
Mean distribution of the density along the radial axis for the sample with the circular slot.

**Figure 13:**
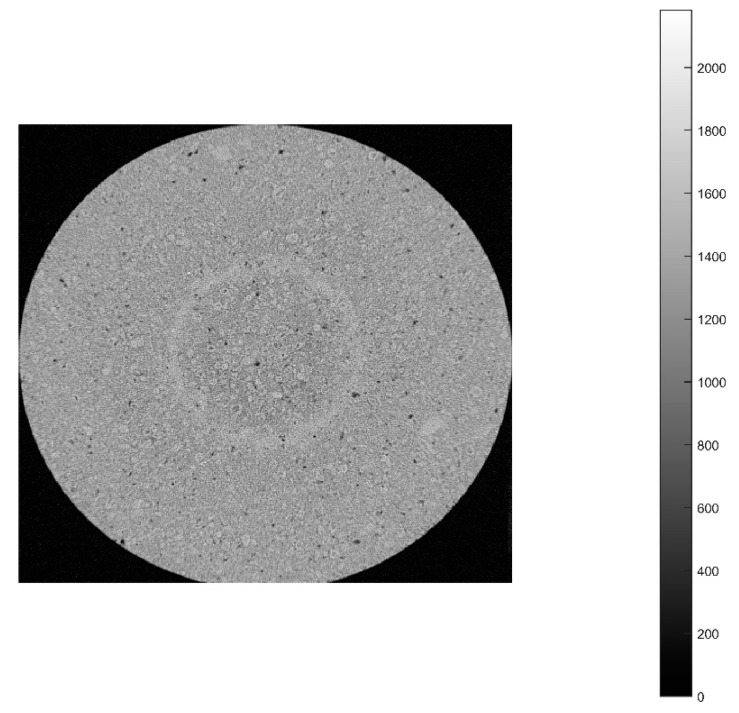
Density profile for a layer located few millimeters above the slot.

**Figure 14:**
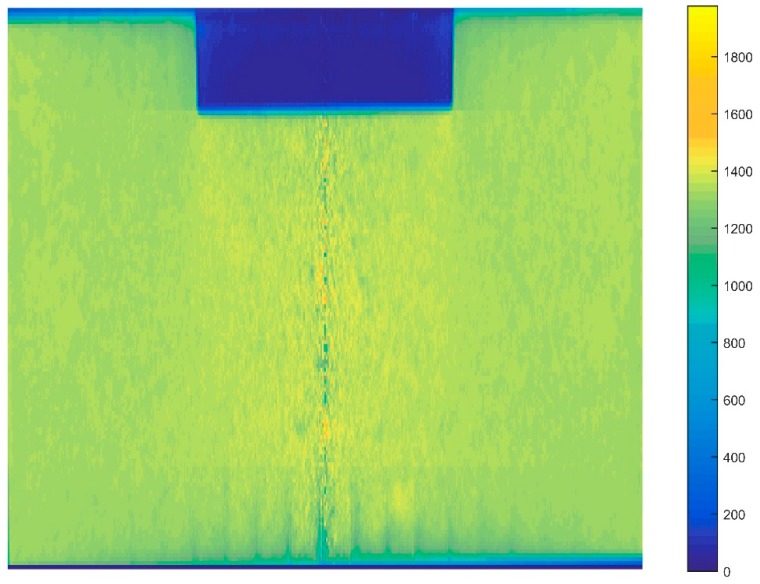
Mean distribution of the density along the radial axis for the sample with the stub hole.

In the aluminum industry, the anode has a complex geometry including slots and stub holes. Consequently, the forming process may lead to significant density gradients. To highlight this trend, a compaction test involving a circular slot placed at the bottom of the mold was carried out (Figure 8). A second compaction test was performed using a simple stub-hole geometry located at the top of the anode paste (Figure 9). Slot and stub-hole geometry dimensions are sketched in Figure 10 and Figure 11. Tests were performed under the same conditions as those for the aforementioned simple compaction experiments. Compacted samples were scanned using X-ray tomography. For more technical details in the application of this technic to carbon materials, the reader is referred to [18,19]. Figure 12 shows the average density obtained for the sample with the circular slot. From a qualitative point of view, we notice that in regions located just above the slot, the anode is more densified. This is also shown in Figure 13, i.e., the density distribution in a plane positioned few millimeters over the slot. In this case, the darker the area, the lower the density. Figure 14 depicts the average density for the sample with the stub hole. Layers located under the stub hole show a relatively higher density than the rest of the sample.

## 4. Inverse Identification and Numerical Simulations

**Figure 15:**
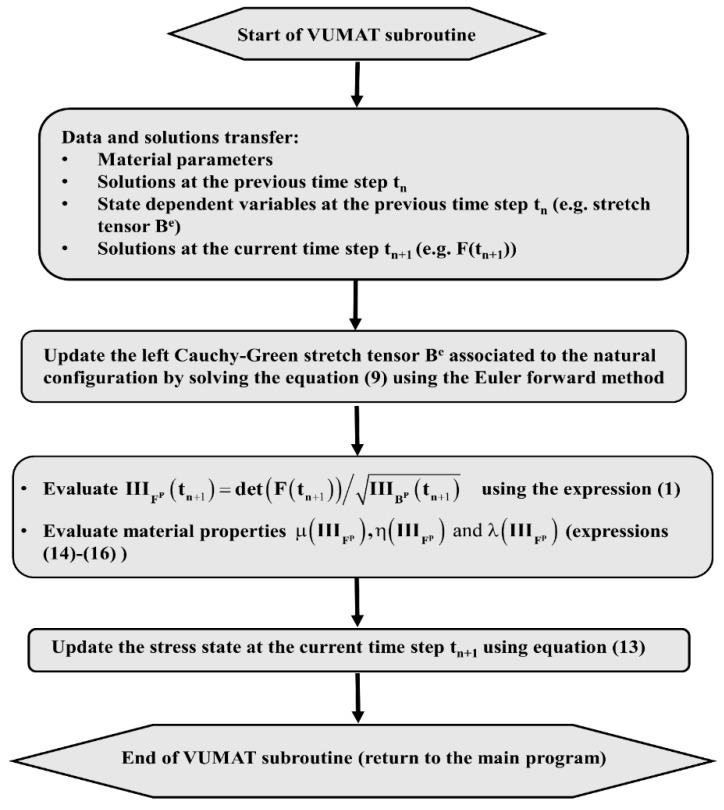
The flowchart of VUMAT subroutine.

**Figure 16:**
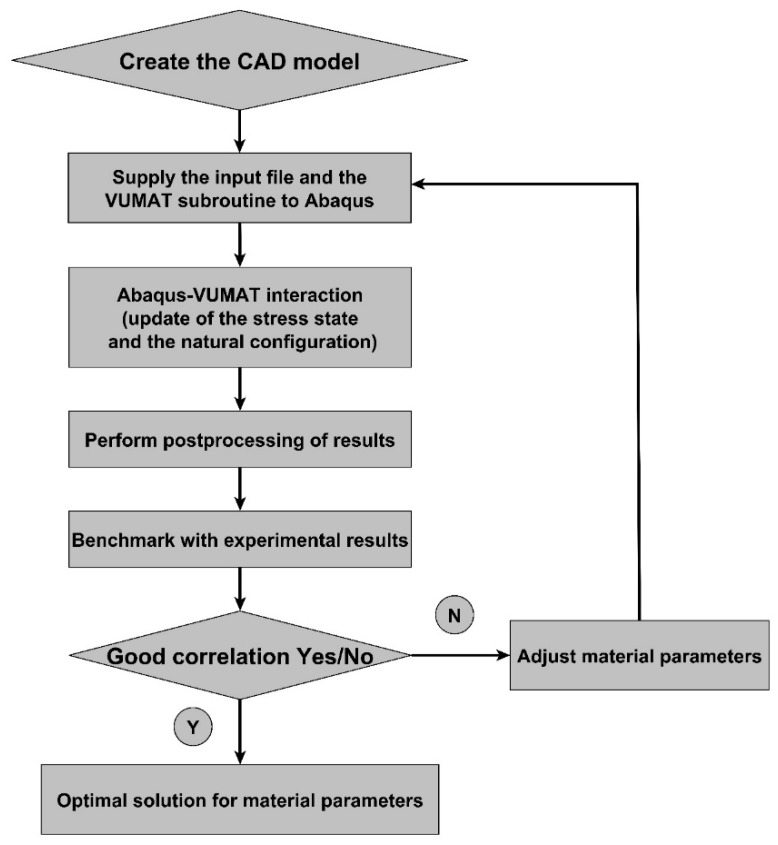
Flowchart used for the inverse identification procedure.

**Figure 17:**
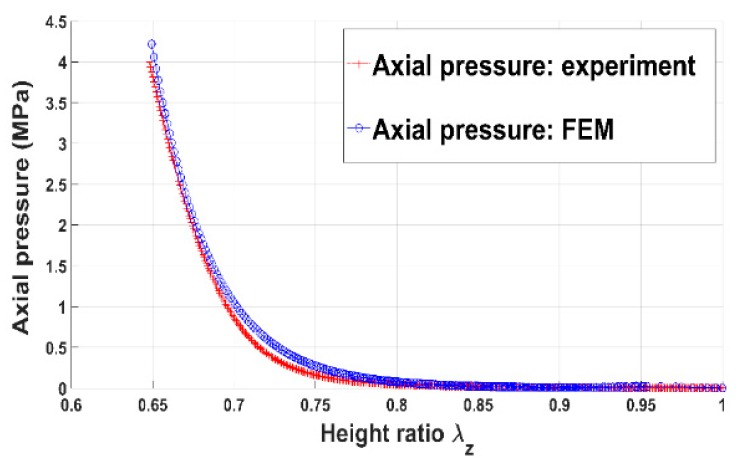
Axial pressure: FEM vs. experiment.

**Figure 18:**
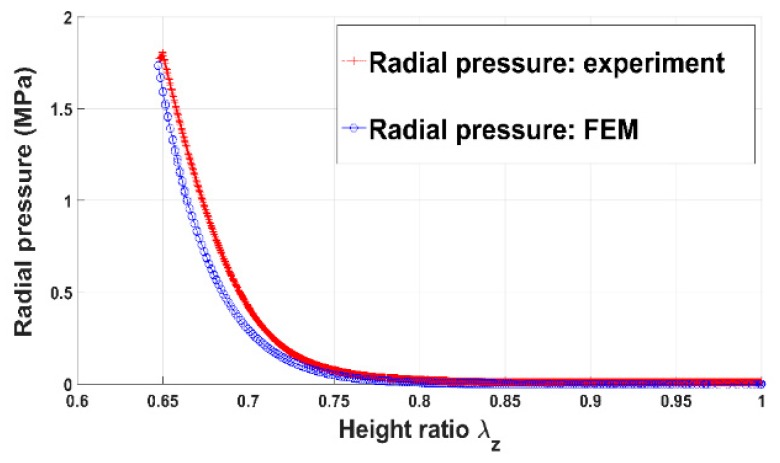
Radial pressure: FEM vs. experiment.

**Figure 19:**
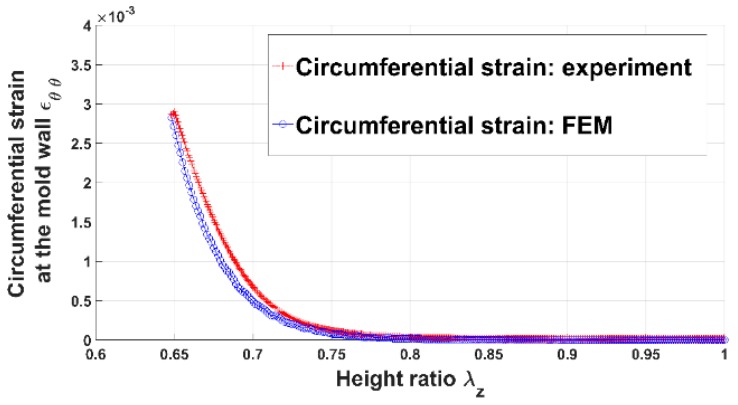
Circumferential strain at the mold wall: FEM vs. experiment.

**Figure 20:**
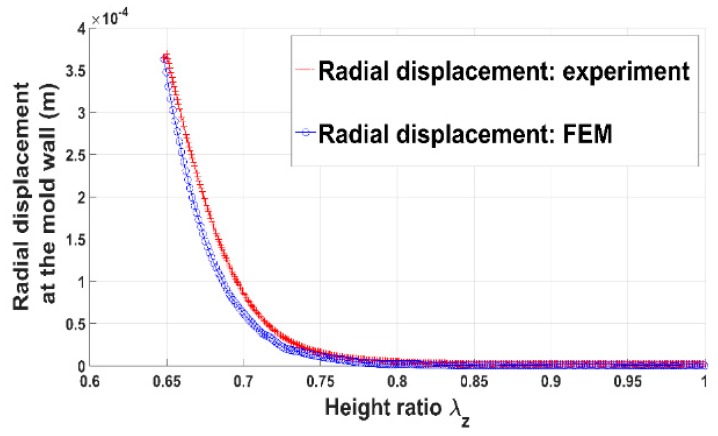
Radial displacement at the mold wall: FEM vs. experiment.

In order to assess the constitutive law predictive capabilities, a user’s material subroutine VUMAT was developed and implemented in the finite element analysis software Abaqus [20]. The implementation of the VUMAT subroutine for the explicit dynamic analysis is illustrated in Figure 15. In a first step, the code was used to identify constitutive law’s material parameters. To this end, the simple compaction test results have been exploited. The flowchart used is depicted in Figure 16. The obtained values for material parameters are reported in Table 2. Figure 17, Figure 18, Figure 19 and Figure 20 show that the model provides good agreement in axial and radial pressures, the circumferential strain, and the radial displacement at the mold wall.

**Figure 21:**
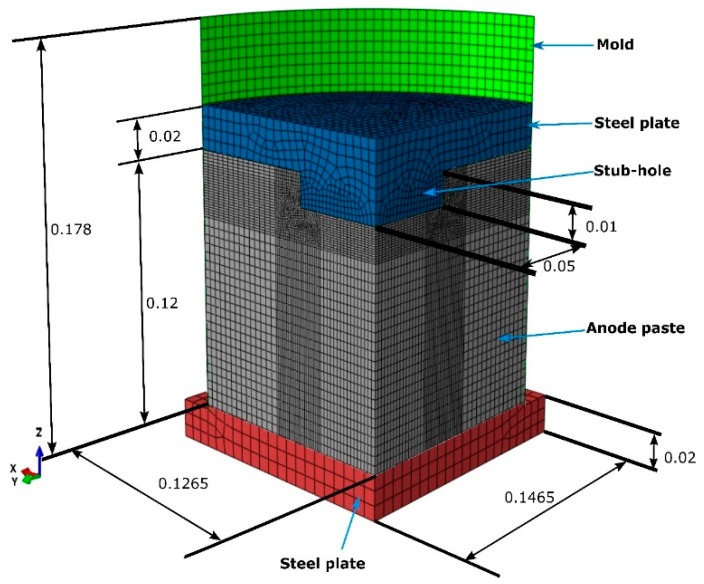
CAD model for the test with the stub hole (m).

**Figure 22:**
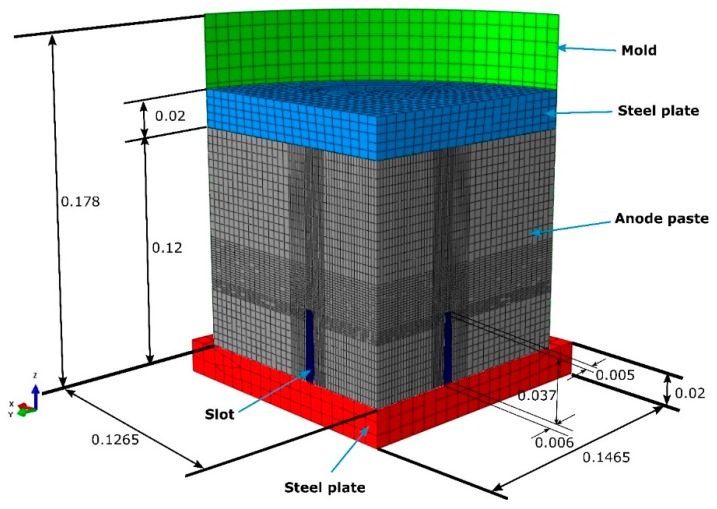
CAD model for the test with the slot (m).

**Figure 23:**
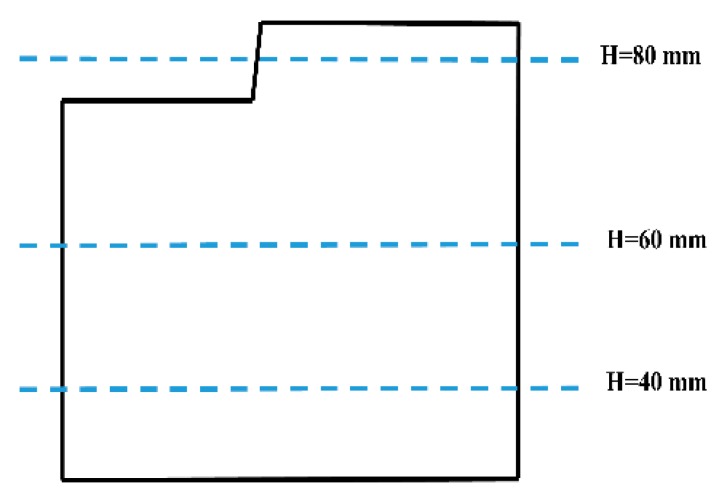
Layers used to compare numerical and experimental results for the stub-hole geometry.

**Figure 24:**
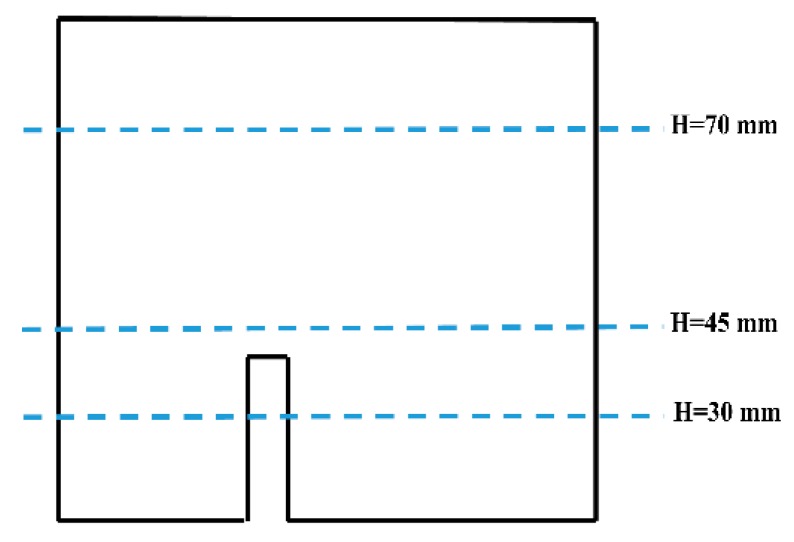
Layers used to compare numerical and experimental results for the slot geometry.

**Figure 25:**
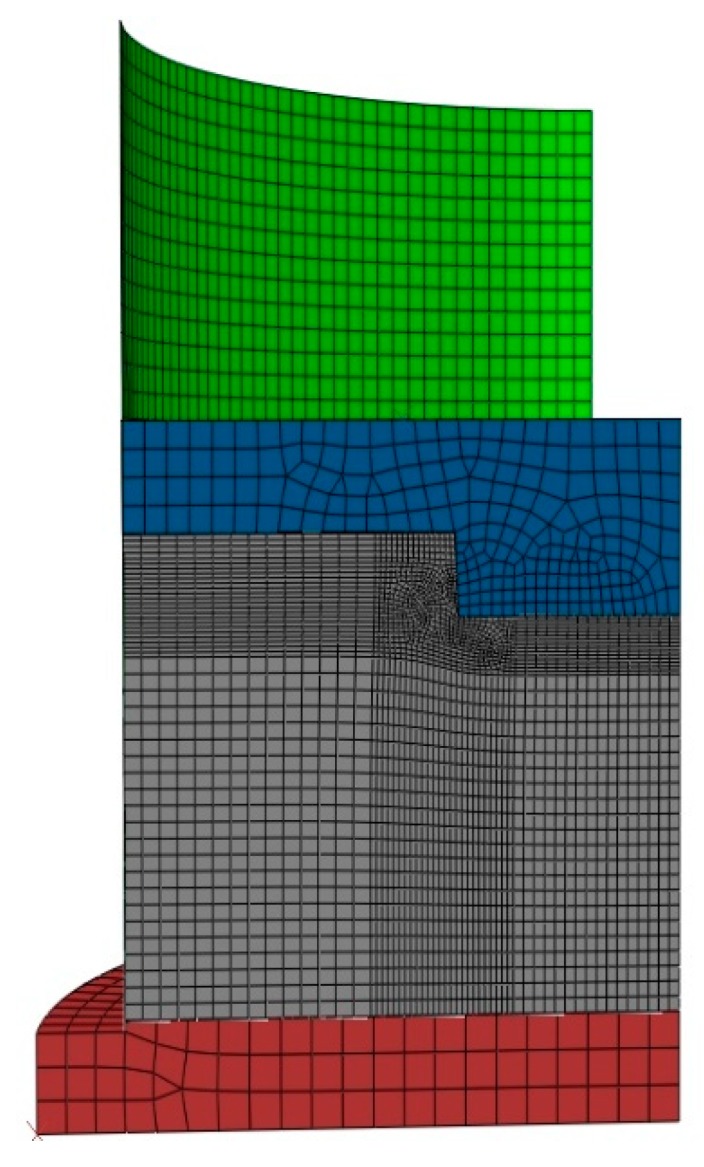
The deformed mesh of the compaction test with the stub hole.

**Figure 26:**
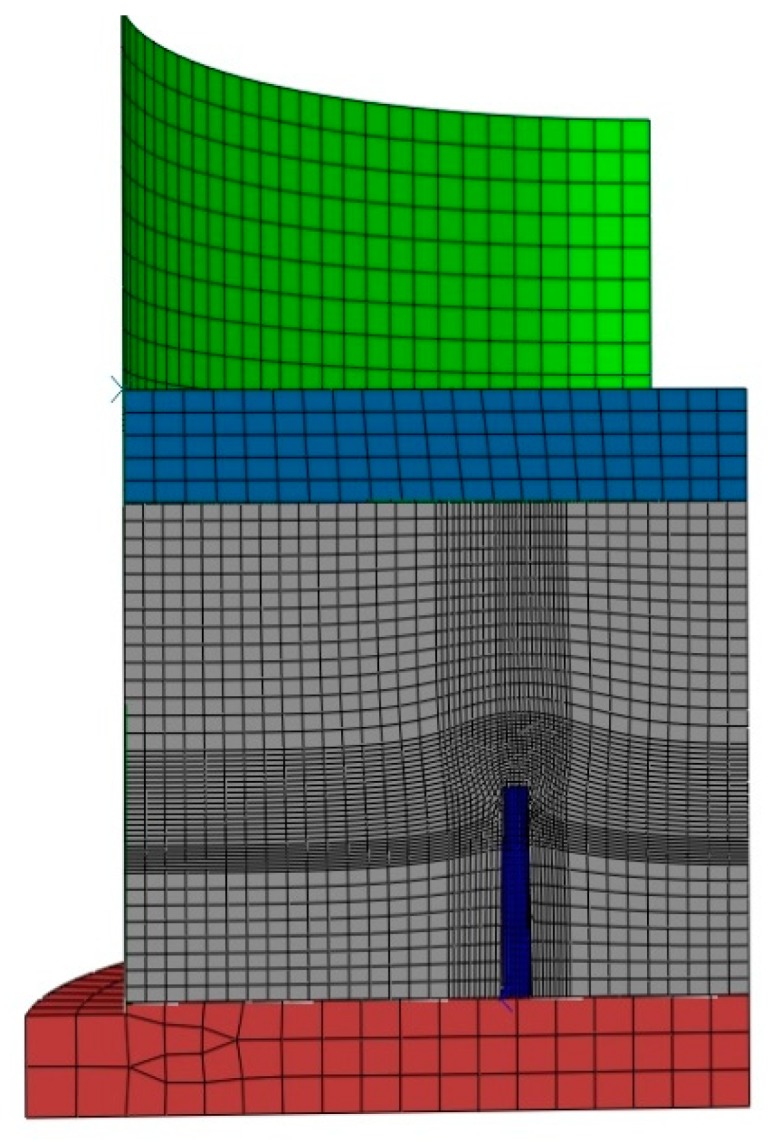
The deformed mesh of the compaction test with the slot.

In a second step, compaction tests involving complex geometries such as the slot and the stub hole were simulated. Figure 21 and Figure 22 show the computer-aided design models used for simulations. Taking into account the symmetry, it should be mentioned that only a quarter of the geometry was simulated. In these simulations, the contact between the mold and the anode was considered through a friction coefficient equal to 0.12. Density profiles predicted by numerical simulations are compared to those computed from experiments using the X-ray tomography. For each geometry, the benchmark between experimental and numerical results is made on three layers (see Figure 23 and Figure 24). Figure 25 and Figure 26 depict the deformed mesh at the end of the simulation of each case.

**Figure 27:**
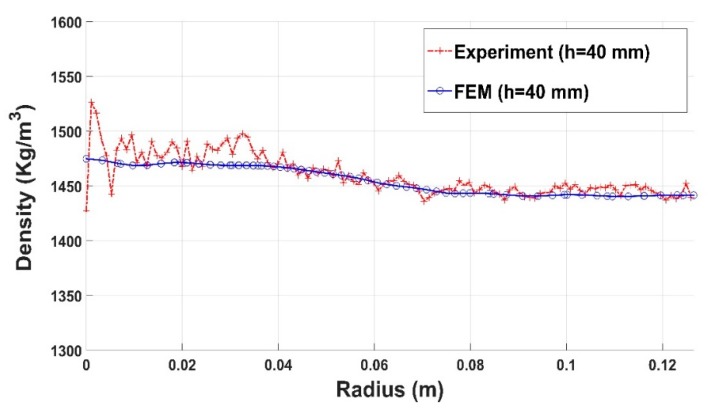
Density profile along the radius (stub hole): experiment vs. finite element result (h = 40 mm).

**Figure 28:**
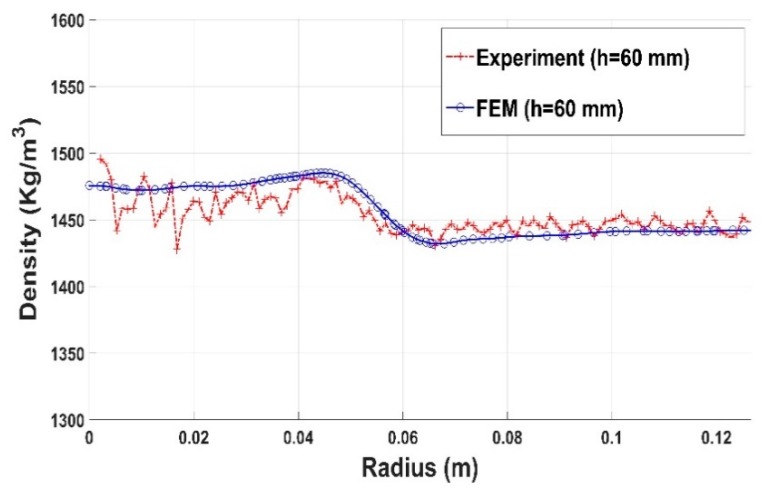
Density profile along the radius (stub hole): experiment vs. finite element result (h = 60 mm).

**Figure 29:**
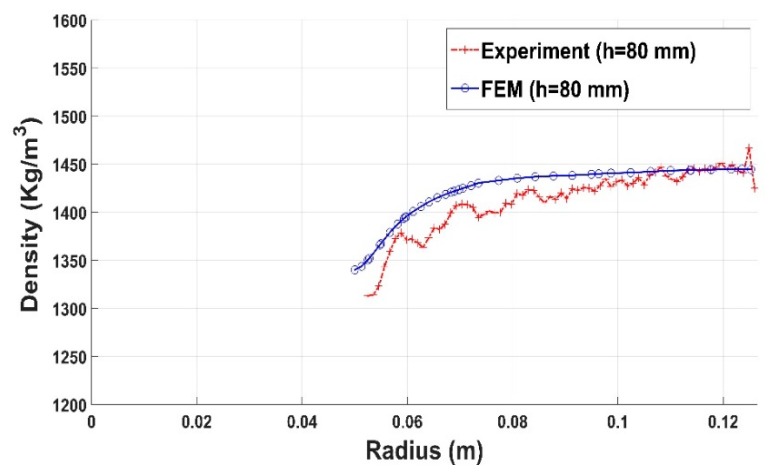
Density profile along the radius (stub hole): experiment vs. finite element result (h = 80 mm).

Figure 27, Figure 28 and Figure 29 show the good agreement between numerical and experimental results for the compaction test using the stub hole. Starting from the mold wall, the density distribution for layers located under the stub hole does not show a significant gradient until we reach the region directly underneath the stub hole where a density gradient is observed (Figure 27 and Figure 28).

**Figure 30:**
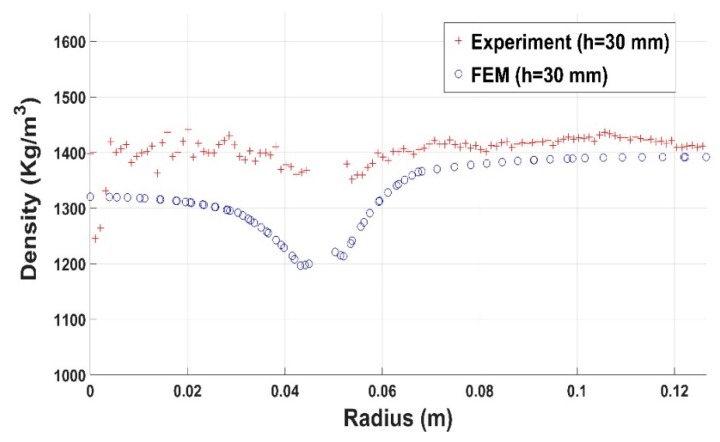
Density profile along the radius (slot): experiment vs. finite element result (h = 30 mm).

**Figure 31:**
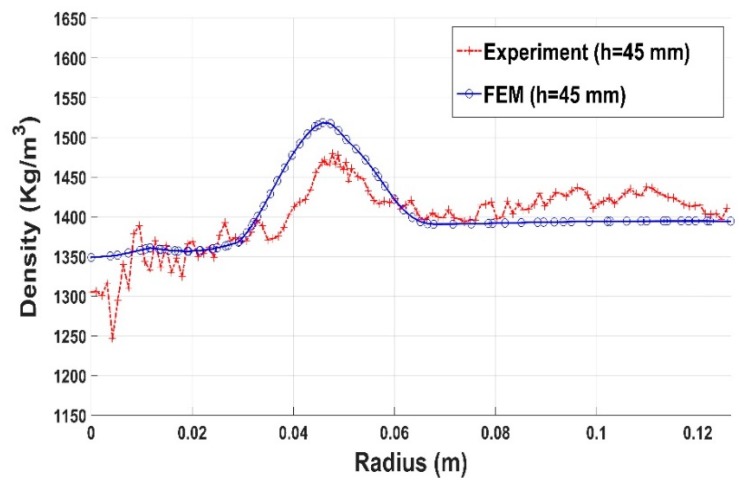
Density profile along the radius (slot): experiment vs. finite element result (h = 45 mm).

**Figure 32:**
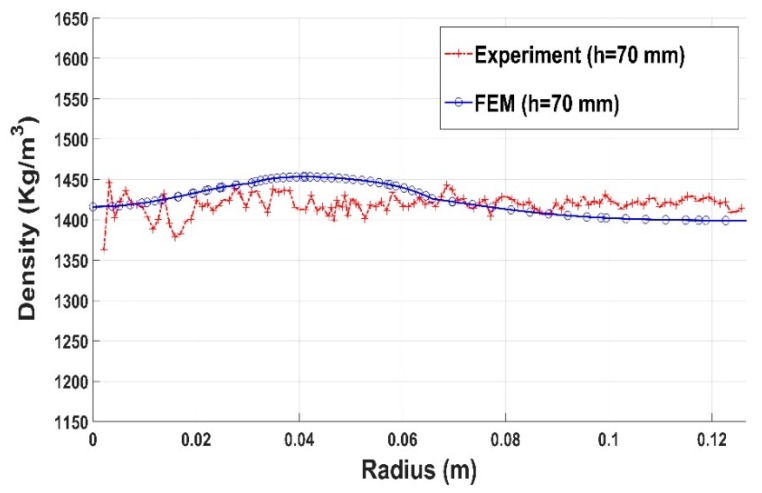
Density profile along the radius (slot): experiment vs. finite element result (h = 70 mm).

Results for compaction test with the slot are shown in Figure 30, Figure 31 and Figure 32. The constitutive law predictions are in conformity with experimental trends. The presence of the slot leads to a significant density variation in the surrounding regions (Figure 30 and Figure 31). However, for regions positioned far from the slot, the density profile seems to be more homogenous (Figure 32).

In light of these results, numerical simulation of the anode compaction process shows that the proposed constitutive law is in good agreement with experimental results, either for simple compaction tests or tests with complex geometries.

## 5. Conclusions

In this work, the compaction behavior of the green anode paste was investigated. For this purpose, a viscoplastic constitutive law, based on the framework of the finite strain theory and the natural configuration concept, was proposed and an experimental setup for the anode compaction process at high temperature was eventually developed. A thin-walled mold was used and the theory of the elastic thin shell was applied to characterize the anode paste radial behavior. Experimental results from simple compaction tests showed the highly nonlinear mechanical behavior of the anode paste and the importance of its radial behavior. The inverse identification procedure showed that the constitutive law reproduces experimental results with good agreement. Furthermore, compaction tests aiming at generation of density gradients were carried out using a stub hole and a circular slot. The specimen’s density was evaluated through X-ray computed tomography. A benchmark with finite element simulation results shows that the constitutive law predicts experimental trends quite successfully.

## Figures and Tables

**Table 1:** Anode paste composition.

Aggregates Size	Content (%)	Mass (g)
−4 + 8	17.9	1072.6
−8 + 14	8.1	487.1
−14 + 28	9.4	565.8
−28 + 48	10.3	619.9
−48 + 100	7.5	447.7
−100 + 200	8.7	521.5
Fines	20.1	1205.4
Pitch	18.0	1080.0
Total	100	6000.0

**Table 2:** Inverse identification results.

μ(.)				η(.)				λ(.)	
$\hat{\mu }$	$\lambda_{1}$	$n_{1}$	$q_{1}$	$\hat{\eta }$	$\lambda_{2}$	$n_{2}$	$q_{2}$	α	β
$1.2\times 10^{4}$	4	−2.8	2	$1.65\times 10^{6}$	0.4	−5.9	1.9	$3.5\times 10^{3}$	0.033
